# Real-Time Monitoring of Interactions between Solid-Supported Lipid Vesicle Layers and Short- and Medium-Chain Length Alcohols: Ethanol and 1-Pentanol

**DOI:** 10.3390/biomimetics4010008

**Published:** 2019-01-22

**Authors:** Shova Neupane, George Cordoyiannis, Frank Uwe Renner, Patricia Losada-Pérez

**Affiliations:** 1 Institute for Materials Research (IMO), Hasselt University, 3590 Diepenbeek, Belgium; shova.neupane@uhasselt.be (S.N.); frank.renner@uhasselt.be (F.U.R.); 2 IMEC vzw. Division IMOMEC, 3590 Diepenbeek, Belgium; 3 Faculty for Industrial Engineering, 8000 Novo Mesto, Slovenia; georgios.kordogiannis@gmail.com; 4 Biomolecular Physics Laboratory, National Centre for Scientific Research “Demokritos”, 15310 Aghia Paraskevi, Greece; 5 Soft Matter Physics Laboratory, Physics Department, Université Libre de Bruxelles (ULB), Campus de La Plaine, CP223, Boulevard du Triomphe, 1050 Brussels, Belgium

**Keywords:** lipid membranes, adsorption, phase transitions, alcohols, microgravimetry, atomic force microscopy

## Abstract

Lipid bilayers represent the interface between the cell and its environment, serving as model systems for the study of various biological processes. For instance, the addition of small molecules such as alcohols is a well-known process that modulates lipid bilayer properties, being considered as a reference for general anesthetic molecules. A plethora of experimental and simulation studies have focused on alcohol’s effect on lipid bilayers. Nevertheless, most studies have focused on lipid membranes formed in the presence of alcohols, while the effect of *n*-alcohols on preformed lipid membranes has received much less research interest. Here, we monitor the real-time interaction of short-chain alcohols with solid-supported vesicles of dipalmitoylphosphatidylcholine (DPPC) using quartz crystal microbalance with dissipation monitoring (QCM-D) as a label-free method. Results indicate that the addition of ethanol at different concentrations induces changes in the bilayer organization but preserves the stability of the supported vesicle layer. In turn, the addition of 1-pentanol induces not only changes in the bilayer organization, but also promotes vesicle rupture and inhomogeneous lipid layers at very high concentrations.

## 1. Introduction

The effect of small foreign molecules as model systems in lipid membranes is relevant to a broad range of biochemical and medical processes where membrane–molecules complexes are involved, namely passive transport through biomembranes, the effect of drugs, and the use of lipid membranes as carriers. Some examples of small foreign molecules include cholesterol, fatty acids, and alcohols, systems that partition differently between the membrane phases and the aqueous environment. The latter have attracted a great deal of attention since they display a wide spectrum of physiological and pharmacological actions such as metabolism, membrane fusion, alcohol toxicity, and general anesthesia [1,2,3,4,5]. In particular, much research has been devoted to the study of lipids–alcohols interactions to elucidate the mechanisms of anesthesia. Alcohol-induced changes in lipid bilayer properties have been extensively studied for a variety of mechanical and thermodynamic properties to shed light on structure–property–function relationships. Examples range from membrane area expansion and decreased bending modulus [6], to changes in molar volumes [7], or channel ionic permeability [8].

As a general picture, short chain alcohols (C < 5) mainly interact with the lipid headgroup, competing with water for hydration sites, while alcohols with a longer chain preferentially bind inserting their tail within the membrane [9,10]. This results in a change in the fluidity of the lipid bilayer reflected by shifts in phase transition temperatures. For instance, the gel to liquid crystalline phase transition temperature of phospholipid-based membranes is strongly dependent on the chain length of alcohols. Short-chain alcohols can promote the formation of a peculiar gel phase, the interdigitated phase [11,12]. For medium-chain length alcohols (C5–C10), the main transition temperature decreases, while it increases again for long-chain ones, the so-called “cut-off effect” [13,14]. From a thermodynamic viewpoint, the effect of an anesthetic is intimately connected with its ability to depress the melting point of lipid membranes, which depends on its solubility in lipid membranes [15]. Extensive experimental evidence supports the above described behavior ranging from differential scanning calorimetry [16,17], light absorbance [18], fluorescence spectroscopy [19], deuterium nuclear magnetic resonance (^2^H-NMR) [20], or X-ray diffraction experiments [21]. However, most experimental studies have focused on lipid membranes formed in the presence of *n*-alkanols, whereas studies on the effect of *n*-alcohols on preformed lipid membranes are rare. Phase contrast microscopy studies have shown that the addition of high ethanol concentration induces shape transformations on giant unilamellar phospholipid vesicles (GUVs) [22]. Compared to GUVs formed in the presence of ethanol, GUVs preformed in water with subsequent ethanol addition exhibit different shape and phase transition temperatures. Furthermore, a recent capacitance study showed that alcohols alter fusion of small liposomes to planar lipid bilayers by enhancing the energy barrier for vesicle fusion, likely through an increase in membrane fluidity due to the presence of alcohol [2].

In the present study, we examined the interactions of supported vesicle layers (SVLs) of dipalmitoylphosphatidylcholine (DPPC) lipid preformed in 4-(2-hydroxyethyl)-1-piperazineethanesulfonic acid (HEPES) buffer with aqueous solutions of alcohols of different length, namely short-chain ethanol and medium-chain length 1-pentanol at the same concentrations. We used quartz crystal microbalance with dissipation monitoring (QCM-D), a label free surface-sensitive technique which, apart from monitoring real-time interactions with SVLs, allows the detection of phase transitions, a useful indicator of membrane organization and structural changes [23,24]. Supported vesicle layers are, together with supported lipid bilayers (SLBs) or tethered lipid bilayers (tSLBs) model biomembrane systems that find applications in bionanotechnology. Specifically, SLVs have attracted attention in pharmaceutical applications as potential drug delivery systems or immunoassays for the detection of small molecules [25,26]. Furthermore, when using QCM-D, the frequency and dissipation changes and phase transition signatures are stronger for SVLs [24]. Alcohol solutions were added when lipids were at the gel phase and the temperature was later changed through the liquid disordered phase. Frequency and dissipation responses reveal that the mechanism of interaction strongly depends on alcohol chain length and concentration. Dramatic differences in layer topography after the addition of ethanol and 1-pentanol were subsequently observed by atomic force microscopy (AFM).

## 2. Materials and Methods

### 2.1. Chemicals and Reagents

Dipalmitoylphosphatidylcholine lipid was purchased from Avanti Polar Lipids (Alabaster, AL, USA) and spectroscopic grade chloroform from Analar (Normapur). HEPES buffer (pH 7.4) consisting of 10 mM HEPES (99%, Thermo Fischer Scientific, FairLawn, NJ, USA) and 150 mM NaCl (≥99.5%, Sigma-Aldrich, Steinhelm, Germany) was utilized for hydration of the dried lipid films. Ethanol (99.96%) was obtained from Analar Normapur VWR International (Fontenay-sous-Bois, France), 1-pentanol (>99%) from Alfa Aesar (Thermo Fischer Scientific, Karlsruhe, Germany). The quantities of lipids were determined gravimetrically using a Sartorius balance (Göttingen, Germany). Alcohol aqueous mixtures were prepared at a concentration of 0.1 and 1.5 M. These two concentrations were selected based on the concentration threshold where interdigitation typically occurs for ethanol-containing membranes [27]. For these concentrations, the relative difference in density, with respect to pure water, is smaller than 1.5%, whereas for viscosity, it is less than 4% for 0.1 M 1-pentanol and less than 15% for 1.5 M ethanol [28,29].

### 2.2. Lipid Vesicle Preparation

Dipalmitoylphosphatidylcholine lipid in powder form was first dissolved in spectroscopic grade chloroform, and the solvent was evaporated under a mild flow of nitrogen in a round bottomed flask. The lipid film was kept under low pressure overnight to remove any remaining solvent. The film was then hydrated with HEPES buffer to 1 mg/mL under continuous stirring in a temperature-controlled water bath at 55 °C (a temperature well-above the melting temperature of DPPC [30]). Small unilamellar vesicles (SUVs) were formed by extrusion through a filter support (Avanti Polar Lipids) with a pore size of 100 nm 25 times following standard procedures [31]. Vesicle effective sizes and polydispersity were determined by dynamic light scattering (DLS) (Zeta Pals, Brookhaven Instruments Corporation, Brookhaven, NY, USA). The obtained average diameters and polydispersity index of the samples used are shown in Table 1.

### 2.3. Quartz Crystal Microbalance with Dissipation Monitoring Measurements

We used QCM-D on a Qsense E4 instrument (Gothenborg, Sweden) monitoring the frequency and dissipation changes ∆*f* and Δ*D*, respectively. Q-sense E4 also enabled heating or cooling temperature scans from 15 to 50 °C. AT-cut quartz crystals with Au coating (diameter 14 mm, thickness 0.3 mm, quoted surface roughness 3 nm, and resonant frequency 4.95 MHz; (Q-sense, Gothenburg, Sweden) were used. The Au-coated quartz sensors were cleaned with a 5:1:1 mixture of Milli-Q water (conductivity of 0.055 S cm^−1^ at 25 °C; Göttingen, Germany), ammonia and hydrogen peroxide (Merk, Darmstadt, Germany), and were ultraviolet (UV)–ozone-treated with a Digital PSD series UV–ozone system (Novascan, Boone, IA, USA) for 15 min, followed by rinsing in Milli-Q water and drying with N_2_. The changes in Δ*f*/*n* (with *n* the overtone number) and in Δ*D* were monitored at five different overtones (from 3rd to 11th). The measurements were made in flow mode with a flow rate of 50 µL/min at 16 °C. The temperature stability at a constant temperature was ±0.02 °C. First, a baseline with pure HEPES buffer was established and afterwards lipid vesicles were injected over the Au-coated sensor chip. After reaching a vesicle layer, the alcohol aqueous mixture was inserted at the same rate for one hour. Following this, the pump was switched off and the ensemble was left to stabilize for several hours. Subsequent temperature scans alternating heating and cooling were performed at a rate 0.4 °C/min, maintaining 30 min of stabilization between successive ramps.

### 2.4. Atomic Force Microscopy Measurements

Atomic force microscopy experiments were performed using a JPK NanoWizard 3 instrument (JPK Instruments AG, Berlin, Germany). Measurements were made in liquid using AC mode. Silicon ACTA-50 tips from AppNano (Mountain View, CA, USA) with a cantilever length of ≈125 µm, spring constant of ≈40 N/m and resonance frequencies of ≈300 kHz (air) and 140 kHz (liquid) were used.

## 3. Results and Discussion

**Figure 1:**
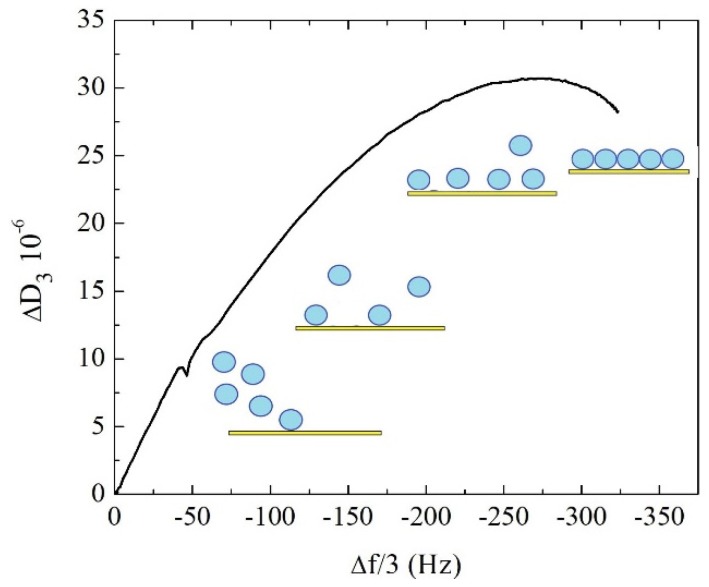
Frequency changes (Δ*D*) vs. dissipation changes (Δ*f*/*n*) plot for the third overtone during adsorption of DPPC vesicles on an Au-coated quartz crystal sensor.

The formation of supported lipid vesicle layers onto Au-coated quartz sensors was monitored in real-time using QCM-D. A suspension of SUVs was injected at 16 °C, with DPPC lipids being in the gel phase. Figure 1 shows a typical Δ*D* vs. Δ*f*/*n* plot for the third overtone where a monotonic increase of both Δ*D* and Δ*f*/*n* indicated continuous adsorption of DPPC vesicles on the sensor until the signal reached a stable value. Gold is indeed well-known to promote the adsorption of intact vesicle layers [32,33]. The maximum in dissipation takes place at intermediate surface coverage where DPPC liposomes experience a “rocking and rolling” motion across the surface. This hydrodynamic effect has been previously observed for DPPC liposomes in the gel phase [34,35]. Dissipation increased with the number of vesicles adsorbed, however, as the surface coverage increased the hydrodynamic interaction between neighboring vesicles precludes rocking and rolling and thus the dissipation of the SVL was reduced.

**Figure 2:**
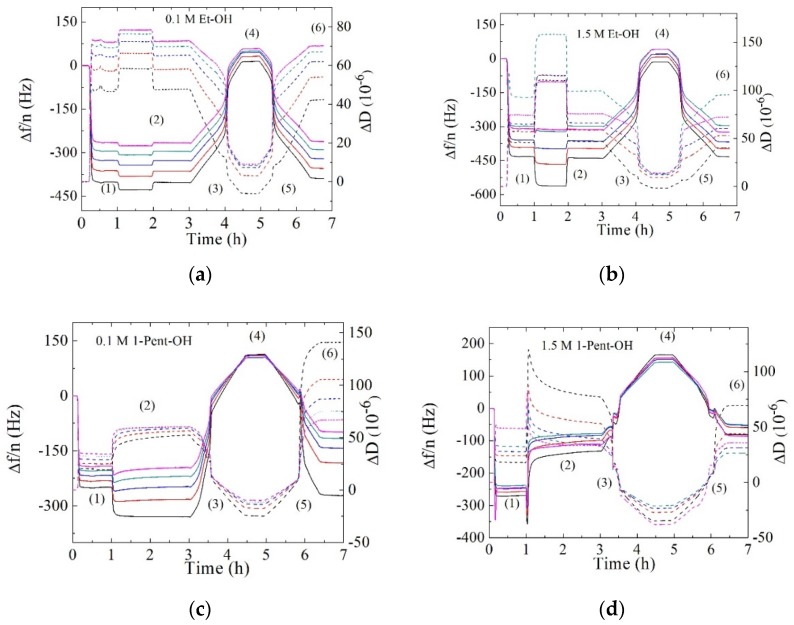
Time dependence of Δ*f*/*n* (solid lines) and Δ*D* (dashed lines) responses during the different stages of a complete experiment. (**a**) DPPC with 0.1 M ethanol (Et-OH); (**b**) DPPC with 1.5 M Et-OH, (**c**) DPPC with 0.1 M 1-pentanol (1-Pent-OH); and (**d**) DPPC with 1.5 M 1-pentanol (1-Pent-OH). Stages: (1) DPPC lipid vesicle addition and layer formation for 1 h; (2) addition of the alcohol system for 1 h and stabilization prior to temperature scans; (3) heating from 16 °C to 50 °C at 0.4 °C/min; (4) stabilization of 30 min at 50 °C before the cooling run; (5) cooling from 50 °C to 16 °C at 0.4 °C/min; and (6) signal plateau at 16 °C. Black lines: third overtone; red lines: fifth overtone; blue lines: seventh overtone, green lines: ninth overtone, pink lines: eleventh overtone.

After an intact SVL was formed, alcohol aqueous mixtures of the concentrations mentioned in Section 2.1 were added at a rate of 50 µL/min in order to examine their dynamic interaction process with DPPC vesicle layers. Figure 2 shows an overview of the time dependence of Δ*f*/*n* and Δ*D* signals for all the measured overtones before and after addition of 0.1 and 1.5 M alcohol aqueous solutions. The relevant stages are denoted by a number in parentheses. During stage 1, vesicles were introduced in the system after a stable baseline in HEPES buffer was reached, leading to large frequency and dissipation shifts with nonoverlapping overtones. The Δ*f*/*n* and Δ*D* values of different overtones provide three-dimensional (3D)-information of the film on the sensor surface. The penetration depth of the harmonic wave was inversely proportional to its frequency, thus higher overtones were more surface-sensitive whereas lower overtones account for changes inside the layer or at the layer–buffer interface. After the formation of the stable vesicle layer, the alcohol aqueous mixture was injected for 1 h and the pump was switched off (stage 2). Stages 3 and 5 consisted of heating and cooling scans at 0.4 °C/min, respectively, with temperature stabilizations in between (stages 4 and 6). The addition of 0.1 M ethanol concentration during stage 2 led to very small changes in Δ*f*/*n* and Δ*D*, namely a slight decrease in Δ*f*/*n* and an increase in Δ*D* (see Figure 2a). For this particular case, the stability of Δ*f*/*n* and Δ*D* signals were sensitive to the solution flow. Nevertheless, after switching off the pump the baseline returned to its original SVL values indicating that vesicle stability was not compromised. After subsequent heating and cooling runs, the baselines of Δ*f*/*n* and Δ*D* at 16 °C remained practically unaltered as compared to their values during the formation of DPPC SVL. As a matter of fact, the Δ*f*/*n* and Δ*D* profiles compared well to the ones obtained for a pure DPPC SVL with no alcohol addition following the same experimental stages (see Supplementary Figure S1). This is in agreement with the fact that ethanol mainly interacts with the lipid headgroup, thus not inducing significant changes in the vesicle layer stability. When the ethanol concentration was increased to 1.5 M, larger shifts of frequency and dissipation were observed during the injection, as a result of the flow during exchange of a solution with a larger difference in viscosity and density. After switching off the pump, the baseline returned to its original SVL values. After heating and cooling scans, the baselines of Δ*f*/*n* and Δ*D* displayed very small changes with respect to their values before the ethanol addition. For both ethanol concentrations, the order of the overtones followed the same trend as during the SVL formation indicating that the vesicle layer remained stable, any mass and energy dissipation changes were very small and took place homogeneously across the lipid vesicles.

Figure 2c,d display the changes ongoing in DPPC supported vesicle layers exposed to solutions of the longer alkyl chain length 1-pentanol. Compared to ethanol, both concentrations used (0.1 and 1.5 M) lie in the “high” concentration range well above the threshold concentration for interdigitation. After addition of 0.1 M 1-pentanol, a decrease in Δ*f*/*n* and an increase in Δ*D* shifts were observed and, unlike for layers exposed to ethanol, the baseline did not return to its original SVL values after switching off the pump. Changes in frequency and dissipation were larger for lower overtones than for higher ones, indicating that interactions with 1-pentanol were predominantly determined by the part of the membrane that was not in contact with the solid substrate. After the heating and cooling scans, Δ*f*/*n* and Δ*D* plateau values differed from their original values. Δ*f*/*n* decreased and Δ*D* increased leading to more dissipative layers. For Δ*D*, the order of the overtones was reversed and changes were more important for lower overtones. For high concentrations of 1-pentanol, the interaction mechanism was quite different. Upon addition of 1.5 M 1-pentanol concentration, a fast decrease and subsequent increase was observed in Δ*f*/*n*, while for Δ*D* the opposite behavior occurred, it first increased abruptly and later decreased at a very slow rate. The mass loss was significant, reaching Δ*f*/*n* values that were two times smaller, i.e., for the third overtone Δ*f*/3 ≈ −134 Hz than the plateau values before 1-pentanol addition, Δ*f*/3 ≈ −272 Hz. Conversely, the dissipation increased dramatically, Δ*D*_3_ ≈ 79·10^−6^, as compared to its original value Δ*D*_3_ ≈ 18·10^−6^. After the heating and cooling scans the decrease in frequency was even larger Δ*f*/3 ≈ −80 Hz and the dissipation decreased slightly to Δ*D*_3_ ~ 69·10^−6^. Unlike pore-forming peptides and long-chain cation ionic liquids [36,37,38], which induce rupture of supported vesicles and formation of rigid planar lipid bilayers, a large concentration of 1-pentanol did decrease the mass in the SVL but not the energy dissipated. The measurements were repeated, and a similar trend was obtained (see Supplementary Figure S2). This rather unique inverse frequency and dissipation trend has been observed recently for membranes exposed to the antimicrobial peptide Ib-AMP-4. This peptide disrupts membrane integrity through a nonlytic mechanism via transient pore formation and membrane resealing. The membrane thus undergoes a dramatic morphological transition where its surface becomes uneven [39]. In our case, elucidating the mechanisms behind such frequency and dissipation changes upon exposure to 1.5 M 1-pentanol concentration was not straightforward and was most likely due to a combination of the large osmotic pressure, the non-negligible change in viscosity of the aqueous alcohol solution compared to pure buffer and the tendency of 1-pentanol to interact with the hydrophobic lipid core. 

**Figure 3:**
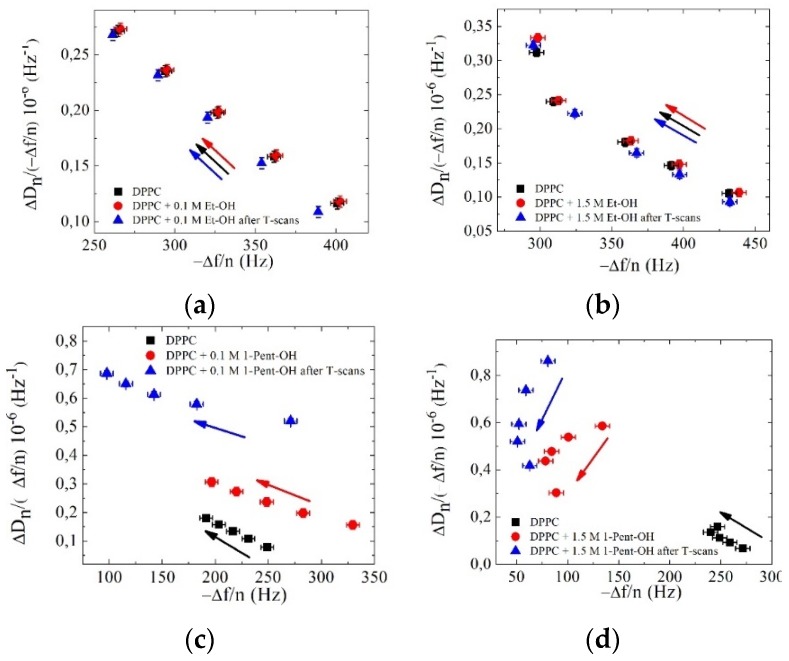
Δ*D_n_*/(−Δ*f*/*n*) ratios as a function of (−Δ*f*/*n*) for different stages of the experiments. (**a**) DPPC + 0.1 M ethanol; (**b**) DPPC + 1.5 M ethanol; (**c**) DPPC + 0.1 M 1-pentanol; and (**d**) DPPC + 1.5 M 1-pentanol. Arrows indicate the sense of increasing overtone frequency. The error bars have been estimated from ≈200 points of the plateau values.

A complementary way to show the changes in the SVL layer after the alcohol solution addition is shown in Figure 3. The shifts in dissipative energy losses over frequency, Δ*D_n_*/(−Δ*f*/*n*), are plotted as a function of the frequency shift. The ratio Δ*D_n_*/(−Δ*f*/*n*) depends both on the size of the liposomes and the surface coverage, increasing with the former and decreasing with the later. This ratio is typically used to estimate the liposome diameter at low surface coverage by extrapolating the linear trend to zero Δ*D_n_*/(−Δ*f*/*n*) value, assuming that for a complete surface coverage the trapped liquid between the adsorbed liposomes ideally occupies voids and is minimal [33,40,41]. Instead, we used the ratio when the SVL was already formed and the surface coverage was large. We have selected points for each overtone at plateau values of Δ*D_n_* and (−Δ*f*/*n*) where a stable vesicle layer was formed: (i) before the addition of the alcohol solution; (ii) after the addition of the alcohol solution; and (iii) after the heating and cooling temperature scans. These plateau values appear as black squares, red dots, and blue triangles in the plots, respectively.

Intact and stable vesicle layers (black squares) showed a linear overtone dependence revealing a homogeneous and acoustically nonrigid vesicle layer. Note that the black square values of the ratio varied slightly from sample to sample since the initial size of the vesicles in the dispersions used differ slightly from sample to sample, as indicated in Table 1.

After the addition of 0.1 and 1.5 M ethanol, the ratio shifted to slightly larger values, while it shifted to smaller values after the heating and cooling temperature scans. In all cases, the linear trend with (−Δ*f*/*n*) was preserved (and thus vesicle layer stability was preserved). Conversely, upon addition of 1-pentanol concentrations significant changes took place. For 0.1 M 1-pentanol, the ratio increased and shifted towards higher frequencies. After heating and cooling scans, a linear trend with a different slope was observed, the ratio increased further and shifted towards lower frequencies, indicating the mass loss events. This result is in agreement with ^1^H-NMR measurements at similar alcohol concentrations which point towards the fact that medium-chain alcohols (C5–C7) increase the permeability of the membrane promoting vesicle lysis or rupture when the main transition temperature is crossed [42]. The addition of the 1.5 M 1-pentanol solution induced shifts towards lower frequencies; however, the order of the overtones was reversed in this case. The high 1-pentanol concentration generated osmotic stress and vesicle deformation, which combined by the 1-pentanol tendency to interact with the hydrophobic lipid core resulted in inhomogeneous mass removal and an asymmetric membrane disruption leading to a highly nonuniform surface structure as will be later seen by AFM images of the surface.

**Figure 4:**
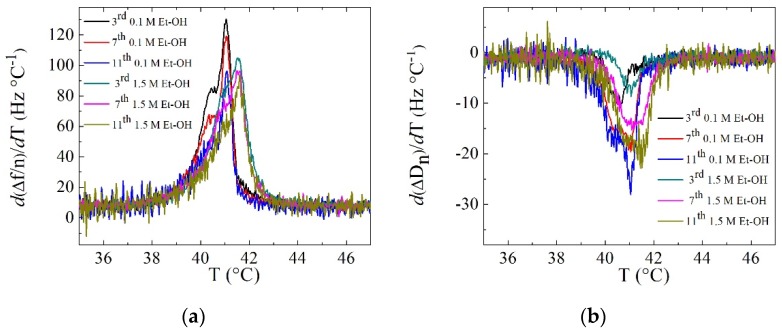
Overtone dependence of (**a**) *d*(Δ*f*/n)/*dT* and (**b**) *d*Δ*D*/*dT* at the main transition of DPPC + 0.1 M alcohol systems and DPPC + 1.5 M alcohol systems upon heating at 0.4 °C/min.

Further insights into the effect of alcohols on supported lipid vesicle layers can be obtained by examining their main phase transition behavior as the temperature is changed. Upon heating lipid bilayers, SVLs change from a thicker and stiffer gel phase to a less stiff liquid disordered one. A temperature-driven phase transition of a SVL is characterized by a nonregular response in both frequency and dissipation shifts. In particular, the first-order derivative of frequency and dissipation curves stand as adequate indicators for the temperature interval where the transition takes place [24,43,44,45]. Figure 4 shows an example of the main transition in DPPC SVLs in the first-order derivatives of Δ*f*/*n* and Δ*D* after the addition of low and high concentration of ethanol upon heating at 0.4 °C/min. The main transition at 0.1 M ethanol occurred at a lower temperature than that for 1.5 M ethanol and the width of both transitions was similar. This result is in agreement with the so-called “biphasic effect”, or the induction of a new phase, the interdigitated gel phase above a threshold concentration. The biphasic effect is characterized by an initial decrease in the melting temperature at low concentrations and above a threshold the interdigitated gel phase is formed, and the melting temperature increases back. For ethanol, the concentration falls around 1.1 M, while for 1-pentanol the threshold concentration is much lower 0.07 M [46,47]. The frequency derivative displays larger features at the transition than the dissipation derivative. The dissipation in SVLs depends on a delicate balance among the stiffness, the thickness of the layers and the presence of hydrodynamic channels [48]. The stiffness and the thickness contributions oppose each other, because stiffer layers dissipate less. However, this competition is counteracted by the presence of hydrodynamic channels both in the gel and the interdigitated gel phase.

**Figure 5:**
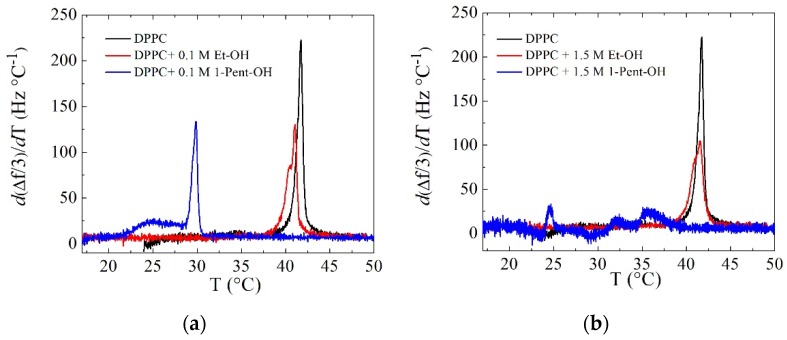
Temperature profiles of the first-order temperature derivative of the frequency shifts upon heating at 0.4 °C/min. (**a**) 0.1 M alcohol concentration, (**b**) 1.5 M alcohol concentration.

Figure 5 shows an overview of the temperature dependence of *d*(Δ*f*/n)/*dT* (ninth overtone) for the SVLs exposed to both concentrations of either ethanol or 1-pentanol upon heating. As expected, 0.1 M 1-pentanol concentration decreased and broadened the main phase transition dramatically when compared to the same concentration of ethanol. Previous calorimetric measurements [46] have also reported a decrease in the main transition temperature although not as pronounced as in this work, probably due to the fact that the transition in our case was accompanied by additional vesicle rearrangements (rupture, changes in shape) in the supported vesicle layers [49]. At 1.5 M 1-pentanol, well above the threshold concentration the frequency derivative displayed a peculiar shape probably due to rupture of liposomes and, as observed for similar concentrations of butanol, the breakdown of the membrane loss of the lamellar structure [17].

**Figure 6:**
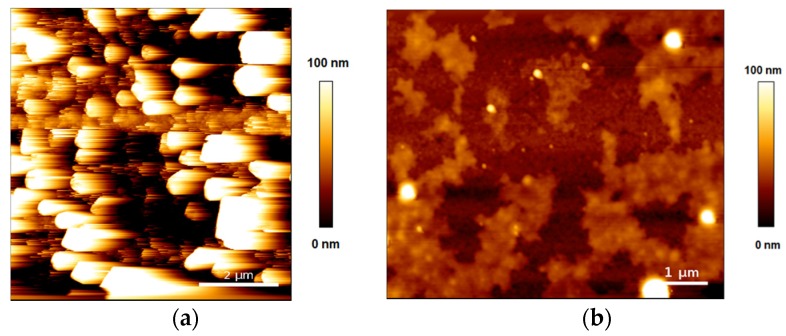
AFM topographic images of the Au-coated sensors after the QCM-D experiments. (**a**) DPPC + 0.1 M ethanol; (**b**) DPPC + 1.5 M 1-pentanol.

After the QCM-D experiments, the sensors were immediately imaged by AFM in AC-mode in liquid. Figure 6 shows an example of the dramatic differences in the topography of the supported vesicle layers after being exposed to ethanol and 1-pentanol (see Supplementary Figure S3 for additional AFM images). Figure 6a shows an Au-coated quartz sensor surface very densely covered with lipid vesicles of thickness ranging from 60 to 150 nm. In turn, Figure 6b shows lipid patches of 10 to 15 nm thickness with large structures of 100 nm and 150 nm entrapped in between the patches. The large vesicle density in Figure 6a makes it very challenging to image soft structures, yet, it is informative regarding the difference in morphology between ethanol and 1-pentanol containing layers.

## 4. Conclusions

The effect of the short-chain alcohols on solid-supported DPPC phospholipid vesicle layers on an Au surface was monitored using QCM-D. Specifically, the stability and integrity of the lipid layers after exposure to the same concentrations of ethanol and 1-pentanol was evaluated in real-time.

When exposed to both 0.1 and 1.5 M concentrations of ethanol solution, preformed DPPC supported vesicle layers displayed minor changes in frequency and dissipation shifts and retained the overtone order. The layers preserved their stability and integrity even after the lipids went through their main phase transition. It was also observed that 0.1 M ethanol concentration decreased the main transition temperature while 1.5 M ethanol increased, in agreement with the formation of an interdigitated gel phase. A different interaction pathway was observed by the addition of 1-pentanol solutions. On the one hand, 1-pentanol at a concentration of 0.1 M adsorbed on the lipid vesicles leading to more dissipative vesicle layers. Furthermore, 0.1 M 1-pentanol induced membrane disorder as inferred from the large decrease in main phase transition temperature. After heating and cooling scans, mass loss of the layer was observed due to partial vesicle rupture. Moreover, the addition of 1.5 M 1-pentanol solution resulted in inhomogeneous mass removal and an asymmetric membrane disruption leading to a highly nonuniform surface structure likely driven by the large osmotic pressure and by non-negligible changes in viscosity of the aqueous solution compared to pure aqueous buffer. Elucidating this mechanism deserves further research efforts for a wider concentration range and for a wider spectrum of *n*-alkanols.

## Figures and Tables

**Table 1:** Hydrodynamic diameter and polydispersity indexes obtained by DLS for the DPPC vesicle dispersions used in this work.

Vesicle Dispersion	Diameter (nm) ^1^	Polydispersity Index
Pure DPPC SUVs used without alcohol addition	114 ± 40	0.14
Pure DPPC SUVs used before ethanol addition	139 ± 55	0.19
Pure DPPC SUVs used before 1-pentanol addition	114 ± 30	0.07

DPPC: Dipalmitoylphosphatidylcholine; SUVs: Small unilamellar vesicles. **^1^** The number of measurements performed per sample is *n* = 5. The data is presented as the mean ± standard deviation.

## Supplementary Materials

The following are available online at http://www.mdpi.com/2313-7673/4/1/8/s1, Figure S1: Time dependence of Δ*f*/*n* (solid lines) and Δ*D* (dashed lines) responses of a pure DPPC supported vesicle layer during the different stages of a complete experiment, Figure S2: Time dependence of Δ*f*/*n* (solid lines) and Δ*D* (dashed lines) responses of a DPPC supported vesicle layer exposed to 1.5 M 1-pentanol during the different stages of a complete experiment, Figure S3: AFM topographic images of the Au-coated sensors after the QCM-D experiments.
