# Supplementary material for: Real-Time Monitoring of Interactions between Solid-Supported Lipid Vesicle Layers and Short- and Medium-Chain Length Alcohols: Ethanol and 1-Pentanol
Source: Biomimetics (Basel). 2019 Jan 22;4(1):8. doi: 10.3390/biomimetics4010008 (PMC6477617; doi:10.3390/biomimetics4010008)
Supplement: Supplementary file 1 [file biomimetics-04-00008-s001.pdf]

Biomimetics **2019**, *4*, 8

Supplementary Materials: Real-Time Monitoring of Interactions between Solid-Supported Lipid Vesicle Layers and Short- and Medium-Chain Length Alcohols: Ethanol and 1-Pentanol

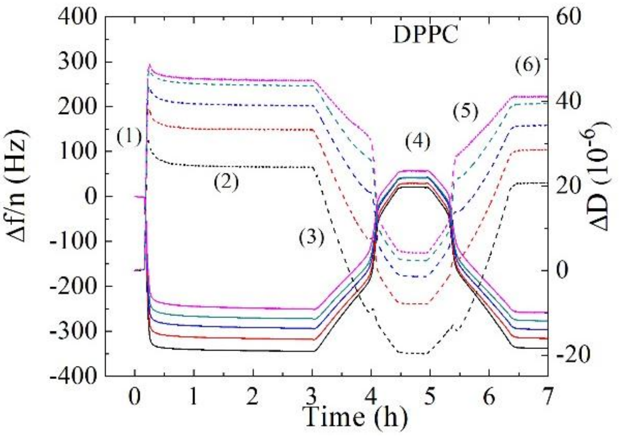

Figure S1. Time dependence of $\Delta f/n$ (solid lines) and ΔD (dashed lines) responses of a pure DPPC supported vesicle layer during the different stages of a complete experiment. Stages: (1) DPPC lipid vesicle addition and layer formation; (2) stabilization.; (3) heating from 16 °C to 50 °C at 0.4 °C/min; (4) stabilization of 30 minutes; (5) cooling from 50 °C to 16 °C at 0.4 °C/min; and (6) signal plateau at 16 °C. Black lines: third overtone; red lines: fifth overtone; blue lines: seventh overtone; green lines: ninth overtone; pink lines: eleventh overtone.

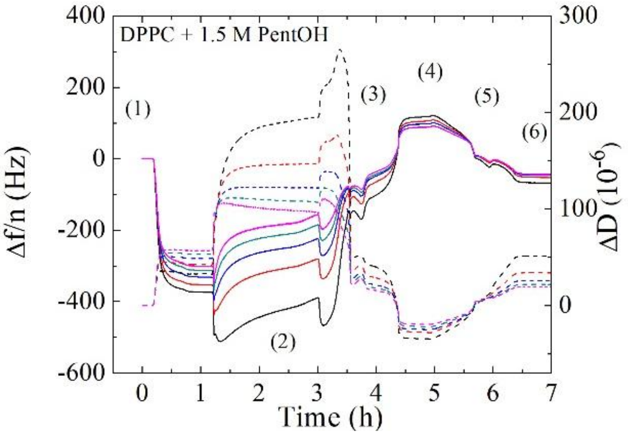

Figure S2. Time dependence of $\Delta f/n$ (solid lines) and ΔD (dashed lines) responses of a DPPC supported vesicle layer exposed to 1.5 M 1-pentanol during the different stages of a complete experiment. Stages: (1) DPPC lipid vesicle addition and layer formation for 1 h; (2) stabilization and addition of 1.5 M 1-pentanol for 1 h; (3) heating from 16 °C to 50 °C at 0.4 °C/min; (4) stabilization of 30 minutes; (5) cooling from 50 °C to 16 °C at 0.4 °C/min; and (5) signal plateau at 16 °C. Black lines: third overtone; red lines: fifth overtone; blue lines: seventh overtone; green lines: ninth overtone; pink lines: eleventh overtone.

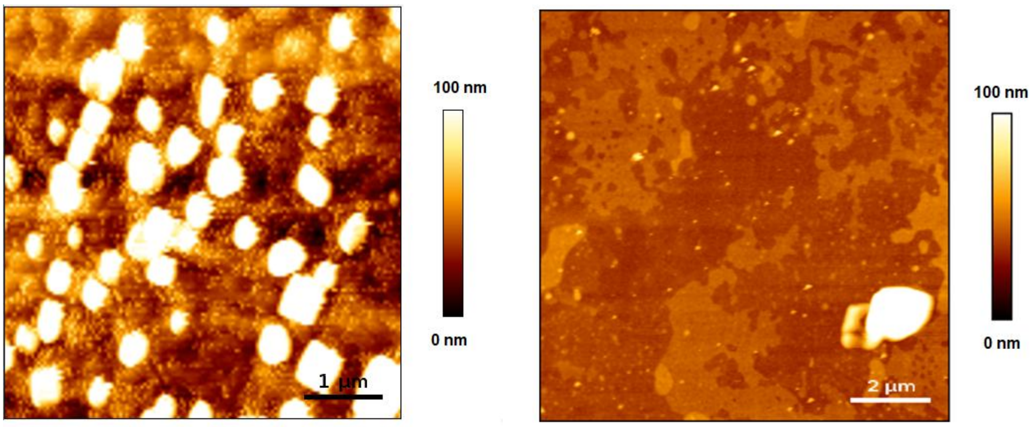

(**a**) (**b**)

Figure S3. AFM topographic images of the Au-coated sensors after the QCM-D experiments. **(a)** DPPC + 1.5 M ethanol; **(b)** DPPC + 1.5 M 1-pentanol.
